# The Effect of the *Tau* Protein on *D. melanogaster* Lifespan Depends on GSK3 Expression and Sex

**DOI:** 10.3390/ijms24032166

**Published:** 2023-01-21

**Authors:** Ekaterina R. Veselkina, Mikhail V. Trostnikov, Natalia V. Roshina, Elena G. Pasyukova

**Affiliations:** 1 Institute of Molecular Genetics, National Research Centre “Kurchatov Institute”, 123182 Moscow, Russia; 2 Skolkovo Institute of Science and Technology, 121205 Moscow, Russia; 3 Vavilov Institute of General Genetics, Russian Academy of Sciences, 119991 Moscow, Russia

**Keywords:** *Drosophila melanogaster*, tau, MAPs, GSK3, the nervous system, synapses, lifespan, sex-dependent traits

## Abstract

The microtubule-associated conserved protein tau has attracted significant attention because of its essential role in the formation of pathological changes in the nervous system, which can reduce longevity. The study of the effects caused by tau dysfunction and the molecular mechanisms underlying them is complicated because different forms of tau exist in humans and model organisms, and the changes in protein expression can be multidirectional. In this article, we show that an increase in the expression of the main isoform of the *Drosophila* melanogaster tau protein in the nervous system has differing effects on lifespan depending on the sex of individuals but has no effect on the properties of the nervous system, in particular, the synaptic activity and distribution of another microtubule-associated protein, Futsch, in neuromuscular junctions. Reduced expression of tau in the nervous system does not affect the lifespan of wild-type flies, but it does increase the lifespan dramatically shortened by overexpression of the *shaggy* gene encoding the GSK3 (Glycogen Synthase Kinase 3) protein kinase, which is one of the key regulators of tau phosphorylation levels. This effect is accompanied by the normalization of the Futsch protein distribution impaired by *shaggy* overexpression. The results presented in this article demonstrate that multidirectional changes in *tau* expression can lead to effects that depend on the sex of individuals and the expression level of GSK3.

## 1. Introduction

Understanding the basis of various pathologies of the nervous system and the associated shortening of lifespan requires the identification of the most significant molecular triggers of related diseases. One of the likely candidates for this role is the *tau* protein, which belongs to the family of microtubule-associated proteins (MAPs), provides microtubule stability in axons [1,2] and plays a role in the control of neurogenesis and synaptic plasticity [3,4]. The impaired function and/or aggregation of the *tau* protein is associated with the development of Alzheimer’s and Parkinson’s diseases [5], as well as a number of diseases combined in the group of tauopathies [6,7].

Of all the possible posttranslational modifications of *tau*, it is assumed that phosphorylation plays the most significant role in the formation of toxic insoluble aggregated forms [8]. Although *tau* activity is normally regulated by phosphorylation, its hyperphosphorylation across a number of epitopes appears to be closely associated with neurodegenerative diseases [9,10]. For example, among the eighty-five potential phosphorylation sites in one of the isoforms of normal human tau, about forty have been predicted to be associated with pathological changes [11,12]. Tau phosphorylation level plays a significant role in maintaining cytoskeleton stability, determining the level of tau association with microtubules and their assembly and stability, as well as axonal transport [13,14,15]. Changes in tau interaction with the cytoskeleton lead to the disruption of the synaptic transmission, behavior, and memory [16,17]. In *Drosophila* models with transgenic human *tau*, it has been demonstrated that learning and memory are affected by Ser238, Thr245, and Ser262 phosphorylation [18,19,20]. In this regard, the identification of key regulators that phosphorylate this protein is essential.

One of the most important proteins involved in tau phosphorylation is the protein kinase GSK3 (Glycogen Synthase Kinase 3). The modulation of GSK3 activity can both enhance and attenuate the effects caused by altered *tau* expression [21,22,23]. The negative effects of the GSK3 overexpression in the nervous system of mice can be reduced by *tau* knockout, while the GSK3 deficiency leads to the decreased phosphorylation of the tau protein [24,25]. In *Drosophila*, the negative consequences of the overexpression of one of the isoforms of human tau are greatly enhanced by the increased expression of the *Drosophila shaggy* (*sgg*) gene encoding GSK3 [26]. It has been demonstrated that this regulation can apparently be mediated by the participation of GSK3 in the noncanonical Wnt pathway [27]. However, a high level of tau phosphorylation is not the only cause of protein toxicity: both increased and decreased GSK3 activity against tau can lead to a pathological phenotype. Apparently, the level of phosphorylation determines the optimal pattern of tau association with microtubules, and its deviation in either direction can have negative consequences [28].

Among the model organisms used to study the molecular basis of neurodegenerative diseases, particularly tauopathies, one of the most popular is the fruit fly *D. melanogaster*. The nervous system of the fruit fly is much simpler than that of mammals, but, at the same time, *Drosophila* neuronal genes have a significant number of orthologs in higher vertebrates [29,30]. Despite differences in the number of repeats responsible for microtubule binding, as well as structural differences in the N-terminal end, the *tau* proteins of the fruit fly and human have a significant, about 66%, level of homology [31]. A similarity has also been demonstrated for the effects of the overexpression of the human and *Drosophila tau* genes: the increased expression of both in the model organism causes abnormalities in the structure of the visual system [32]. Nevertheless, there seem to be nonoverlapping functions between the *Drosophila* and human proteins. Although the increased expression of both variants studied in [32] resulted in similar eye changes, only the overexpression of the *Drosophila* protein under the conditions of the increased expression of par1 gene resulted in lethality. In the same paper, it was demonstrated that the overexpression of the human and fly *tau* proteins in the *Drosophila* genetic background has different effects on the proteome. It is also known that the complete replacement of the *Drosophila tau* gene with the human one affects some functions of the nervous system [33]. Differences in the phenotype caused by changes in the expression of the *Drosophila tau* gene and the human *tau* gene can be caused by differences in the species-specific structure and the function of the protein isoforms. Even different isoforms from the same species can differ functionally. In *Drosophila*, the activation of transgenes corresponding to the human *tau* gene with three or four microtubule-binding motifs affects the nervous system function in different ways [34]. Differences in the effects of human and fruit fly tau proteins can also be explained by differences in the number of microtubule-binding domains. The *Drosophila* tau has five of these, not three or four like the human one. The fifth repeat, which is absent in the human protein, is highly homologous to the *C. elegans* tau [31]. Differences in the structure of the human and fruit fly tau may play a role not only in the maintenance of the basic functions of the protein but also in its interaction with the main regulators.

The identification of risk factors for the development of neurodegenerative diseases allows their causes to be figured out and ways to combat them to be found. The analysis of genetic factors allows to understand the molecular basis of hereditary forms of diseases, and the frequency of alleles associated with the manifestation of the pathologies of the nervous system characterizes the premature death factor burden in the human population. The influence of genetic factors strongly depends on gender and age [35,36,37]. Age is most strongly associated with an increased risk of neurodegenerative diseases as well as with impaired proteostasis, mitochondrial dysfunction, and other changes associated with neurodegeneration [38,39,40]. It has been shown that the level of stress induced in the nervous tissue during aging is dependent on sex and the interaction of sex hormones with specific receptors in the nervous system [41]. Sex differences in the nervous system can be explained by the sex-chromosome dosage effect [42]. It is known that the risk of certain neurodegenerative diseases is higher in women, and in women with moderate cognitive impairment, the tau protein has a wider connectivity network between different brain regions [43,44]. In *Drosophila*, a number of studies have also demonstrated the role of sex, in particular, sex-dependent differences in *tau* expression levels in controlling the pathologies of the nervous system. For example, it has been shown that male and female transcriptomes differ after traumatic brain injury in *Drosophila*, associated with a significant risk of developing neurodegenerative pathologies [45]. Decreased *tau* expression affected motor function and transcription in a sex-dependent manner, but not all neurological pathologies that appeared after brain injury correlated with *tau* expression levels [45].

The effects of tau on the nervous system are complex and determined by a set of isoforms and interactions with other proteins, which, in turn, may depend on gender and other factors. This creates the need to analyze the functional role of individual tau isoforms and their interaction with the individual isoforms of partner proteins with regard to sex and age. Given the close relationship between the decline of the nervous system and aging, it is also important to understand how changes in the expression of individual *tau* isoforms affect lifespan.

In this work, we studied the effect of the increased and decreased expression of the *D. melanogaster* tau in neurons on lifespan and the properties of the nervous system. We demonstrated that the overexpression of the major tau isoform in the nervous system, which includes all four microtubule binding sites present in the human tau, affected the lifespan of females but not of males, and we did not find any dependence of this effect on the GSK3 protein kinase. In contrast, the effect of the overexpression of the major tau isoform on synaptic activity in females was observed only if the GSK3 target sites were changed and, consequently, the interaction of tau with the protein kinase was presumably disrupted. We showed that *tau* knockdown, similar to gene deletion [46], had no effect on lifespan, but in females it mitigated the negative effect of GSK3 overexpression on survival. These results suggest an interaction between *tau* and GSK3 in controlling lifespan. The knockdown of the *tau* gene affecting most tau isoforms also resulted in the restoration of the distribution of the MAP-related Futsch protein in neuromuscular junctions in females against the background of GSK3 overexpression. Our results may indicate a role for the mutual compensation of the functions of different MAPs, not only in controlling the properties of the nervous system but also in controlling lifespan. Taken together, all our findings indicate that the effects of tau on the nervous system and lifespan are not always correlated, but both depend on sex and a complex interaction between *tau* and GSK3.

## 2. Results and Discussion

To assess the effect of increased and decreased tau expression in neurons on lifespan and the properties of the nervous system, we used tau overexpression and knockdown. Fourteen tau isoforms are currently predicted (https://flybase.org/reports/FBgn0266579, accessed on 20 January 2023). We cloned and overexpressed cDNA corresponding to the *RA* transcript of *tau*, encoding the major neuronal tau isoform [46], which includes all four microtubule binding sites present in the human tau. To better understand the interaction between tau and GSK3, we also cloned and overexpressed the major isoform with mutations affecting three amino acids that are phosphorylated by GSK3. These sites for mutagenesis were selected based on the comparison of *Drosophila* and human tau: in humans, the matching phosphorylation sites were shown to be associated with neuronal pathologies [47,48,49]. To mask the selected phosphorylation sites, serine and threonine were replaced with alanine, which does not greatly alter the protein structure and cannot be phosphorylated. For tau knockdown affecting all tau transcripts except RE (JBrowse 3R:27636471..27660425 (flybase.org)), we used the line obtained from Bloomington Drosophila Stock Center USA), which was also used in [50], where a significant decrease in protein amount as a result of knockdown was shown.

### 2.1. Tau Affects Drosophila Life Span

#### 2.1.1. Overexpression of the *RA* Transcript of the *Tau* Gene Reduces the Lifespan of Females

**Figure 1:**
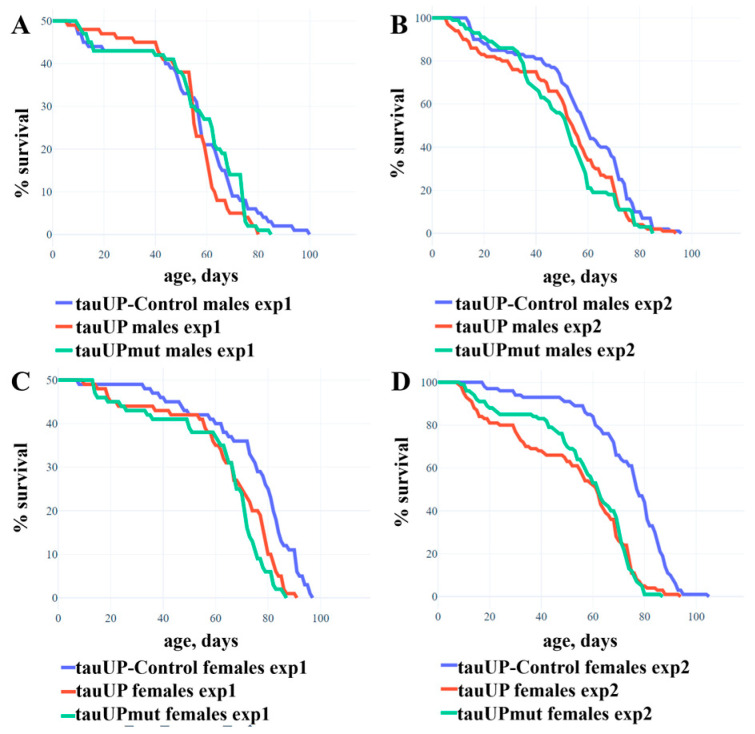
Increased expression of the normal and mutant *RA* transcript of the *tau* gene leads to a significant decrease in the lifespan of females and a minimal or insignificant effect on the lifespan of males. (**A**,**B**)—Experiments 1 and 2 with males. (**C**,**D**)—Experiments 1 and 2 with females. A detailed description of the genotypes is given in the Materials and Methods section. Statistical analysis of the data is given in Supplementary Table S1.

To evaluate the effect of the increased expression of the *D. melanogaster tau* gene on lifespan, we used the transgenic lines with cDNA insertions encoding the normal *RA* transcript of the fruit fly *tau* gene (the tauUP line) or the *RA* transcript with mutations leading to amino acid substitutions in phosphorylation sites serine 103, threonine 151, and serine 343, which are targets of GSK3 protein kinase (the tauUPmut line). The tauUP-Control line with the same genetic background was used as a control. Transgene activation was induced in the nervous system at all stages of development using the pan-neuronal driver line NS. Trait evaluation was performed in two experiments, with sample sizes of 50 (Figure 1A,C) and 100 (Figure 1B,D) individuals per genotype/sex, respectively. In the first experiment, the overexpression of both *tau* transgenes resulted in a significant decrease in the mean lifespan of females (*p* = 0.006, Figure 1C) but not of males (*p* = 0.388, Figure 1A). In the second experiment with a larger sample, the negative effect was stronger in females (*p* < 0.001, Figure 1C) and a weak but statistically significant negative effect was detected in males (*p* = 0.016, Figure 1B). Such fluctuations between the results of repeated experiments are quite typical when the effects are virtually absent: in several subsequent experiments, the differences turned out to be significant or not at random [51]. In none of the experiments did the lifespan of individuals with the overexpression of the normal and mutant *tau* transgene differ significantly from each other.

Thus, we found that the overexpression of both normal and mutant *RA* transcripts of the *tau* gene significantly reduced the lifespan of females but had no or very little effect on male survival. This result indicates a specific and sex-dependent role of a particular *tau* gene transcript in the nervous system. Although the second experiment revealed a weak effect of the increased expression of the *RA* transcript on the lifespan of males, our results generally indicate its more important role in the nervous system of females. This may be due both to the sex-dependent function of the *RA* transcript itself and to the specifics of the posttranscriptional regulation of tau protein activity in the individuals of different sexes. These findings take on added importance in view of the fact that Alzheimer’s disease is significantly more common in women [52,53] and may indicate the possibility of a sex-dependent function of certain tau isoforms in humans.

Despite the proven role of GSK3 in phosphorylation and, consequently, the regulation of tau protein activity, we observed the same decrease in lifespan in females overexpressing both the normal *RA* transcript and the *RA* transcript with mutations masking epitopes for GSK3 phosphorylation. In order to gain a deeper understanding of this issue, we performed additional experiments.

#### 2.1.2. Overexpression of the *RA* Transcript of the *Tau* Gene Does Not Affect the Reduction in Lifespan Caused by the Overexpression of the *RB* Transcript of the *Shaggy* Gene

**Figure 2:**
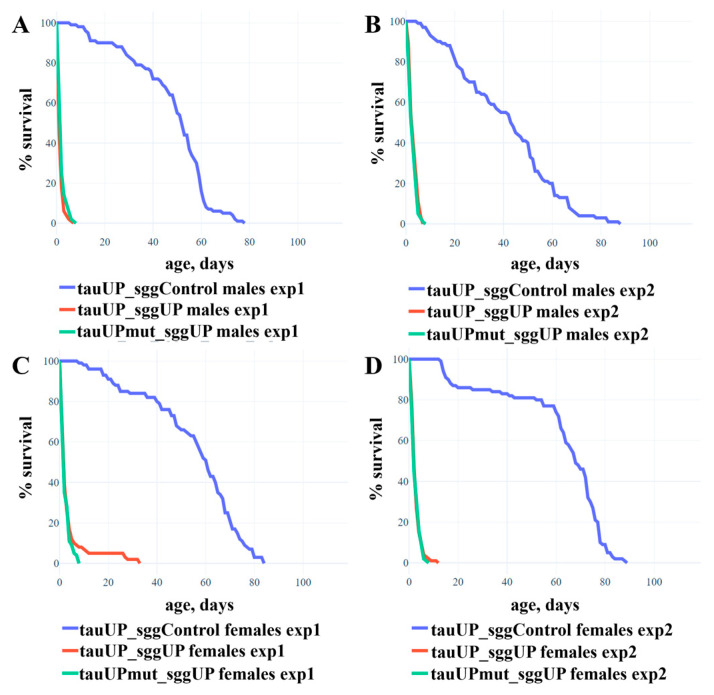
Co-activation of transgenes that increase the expression of the normal or mutant *RA* transcript of the *tau* gene and the *RB* transcript of the *shaggy* gene reduces the lifespan of males and females. (**A**,**B**)—Experiments 1 and 2 with males. (**C**,**D**)—Experiments 1 and 2 with females. A detailed description of the genotypes is given in the Materials and Methods section. Statistical analysis of the data is given in the Supplementary Materials Table S2.

In [54], using the sggUP and sggUP-Control lines and the pan-neuronal driver elav, we demonstrated that the overexpression of the *RB* transcript of the *shaggy* gene encoding the GSK3 protein kinase of *D. melanogaster* in the nervous system severely reduces the lifespan of males and females (mean lifespan of males is ~2 days, females ~6 days). Since the tau protein is a target of GSK3, we can assume that a simultaneous increase in *tau* and *shaggy* expression will result in a non-additive enhancement of the effect in females, i.e., lethality. However, if the sites at which GSK3 phosphorylates tau are altered, the interaction of the proteins will be disrupted and there will be no enhancement of the effect. To test these assumptions, we used individuals whose genome contained two transgenes: one providing the increased expression of the normal or mutant *RA* transcript of the *tau* gene and the other providing the increased expression of the *RB* transcript of the *shaggy* gene (the lines tauUP_sggUP and tauUPmut_sggUP). The tauUP_sggUP-Control line with the same genetic background was used as a control. The transgene activation was induced in the nervous system at all the stages of development using the pan-neuronal driver line NS. In two repeated experiments, males and females with two activated transgenes were viable, and although their lifespan was greatly reduced compared to controls, it did not differ from the lifespan of males and females with an overexpression of the *shaggy* gene alone [54] (Figure 2; the mean lifespan of males was ~2 days, of females ~3 days). Differences in lifespan between males (or females) with an increased expression of the normal *tau* gene and *shaggy* gene and males (or females) with an increased expression of the mutant *tau* gene and *shaggy* gene were not found.

The result is consistent with the additive, independent effect of *tau* and *shaggy* on lifespan. Indeed, in males, we found no significant effect on the lifespan of the *tau* transgene overexpression itself, nor did the overexpression of this transgene change the effect of *shaggy* overexpression. In females, a slight decrease in lifespan as a result of the overexpression of the *RA* transcript of the *tau* gene was undetectable against a dramatic decrease in lifespan as a result of the overexpression of the *RB* transcript of the *shaggy* gene (~3 days versus ~6 days).

Overall, our experiments suggest that, while affecting lifespan separately, tau and GSK3 do not interact with each other. This result can be explained by several reasons. It is possible that, in the lifespan control pathway, tau is not a target of GSK3 at all. This assumption was confirmed both by the fact that the effect of *tau* and *shaggy* gene overexpression on lifespan was additive and by the fact that blocking the GSK3 target sites in the tau protein did not affect the observed effects. Perhaps tau is not targeted by GSK3 only at some stages of development, such as embryonic, when the expression of a number of genes, including *shaggy*, has been shown to be important in controlling the lifespan [54,55,56]. In part, our results can be explained by the fact that the level of phosphorylation of the mutant sites does not affect those properties of the tau isoform encoded by the *RA* transcript that are essential for its participation in lifespan control. Finally, in our experiments, we upregulated the expression of individual transcripts of both *tau* (*RA* transcript) and *shaggy* (*RB* transcript), so our results may indicate complex and non-obvious interactions between the individual forms of the respective proteins.

#### 2.1.3. Knockdown of the *Tau* Gene Has No Effect on the Lifespan of Males and Females

**Figure 3:**
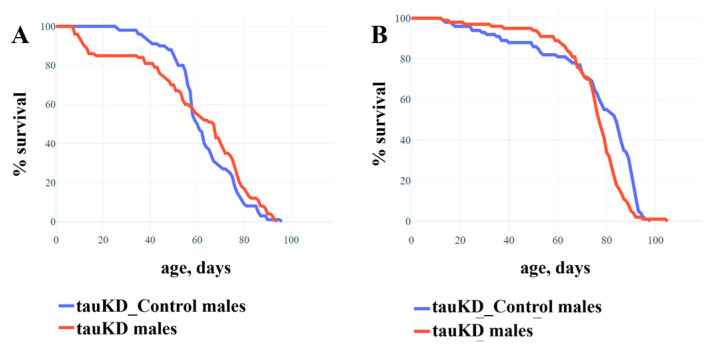
Decreased expression of the *tau* gene has no effect on the lifespan of males and females. (**A**)—Experiment with males. (**B**)—Experiment with females. A detailed description of the genotypes is given in the Materials and Methods section. Statistical analysis of the data is given in the Supplementary Materials Table S3.

It has previously been demonstrated that, in *D. melanogaster*, a decrease in tau function caused by the excision of certain exons, presumably encoding microtubule binding domains from the gene, has no effect on lifespan [46]. In this study, using the line tauKD, we evaluated the lifespan of males and females in which tau function was reduced as a result of transgene activity, causing the RNAi knockdown of most of the gene transcripts. The tauKD-Control line with the same genetic background was used as a control. Transgene activation was induced in the nervous system at all stages of development using the pan-neuronal driver line NS. Our data confirmed that a decrease in *tau* gene expression did not lead to a decrease in lifespan in either males or females (Figure 3A,B). It is worth noting that despite the statistical significance of the result in females (*p* = 0.011), there was no difference in mean lifespan between control and experimental cohorts (tauKD_Control ~75 days, tauKD ~74 days).

The absence of the effect of the *tau* gene dysfunction on survival revealed in this and in the earlier paper [46] may be related to the complex organization of this gene in *D. melanogaster* (http://flybase.org/reports/FBgn0266579, accessed on 20 January 2023). Fourteen *tau* transcripts have been described, and the functions of some of these may have been preserved when certain exons were excised, as described in [46]. As for the gene knockdown, according to the manufacturer, it does not affect all transcripts. For example, the *RE* transcript was not affected by either the exon excision or the RNAi knockdown and could compensate, at least with respect to lifespan, for the effect of the absence of other transcripts. In the future, a study of the functional specificity of minor *tau* transcripts could help to better characterize the function of the gene as a whole.

#### 2.1.4. Knockdown of the *Tau* Gene Positively Affects the Lifespan of Females with the Overexpression of the *RB* Transcript of the *shaggy* Gene

The *tau* gene knockdown used in this work did not cause changes in the lifespan of males and females. This suggests that, since the tau protein is a target of GSK3, a decrease in tau caused by the knockdown of the *tau* gene would attenuate the severe negative effect of *shaggy* overexpression on lifespan. To test this assumption, we used individuals from the tauKD_sggUP line whose genome contained two transgenes: one provided the *tau* knockdown and the other provided the increased expression of the *RB* transcript of the *shaggy* gene. The tauKD_sggUP-Control line with the same genetic background was used as a control. Transgene activation was induced in the nervous system at all stages of development using the pan-neuronal driver line NS.

**Figure 4:**
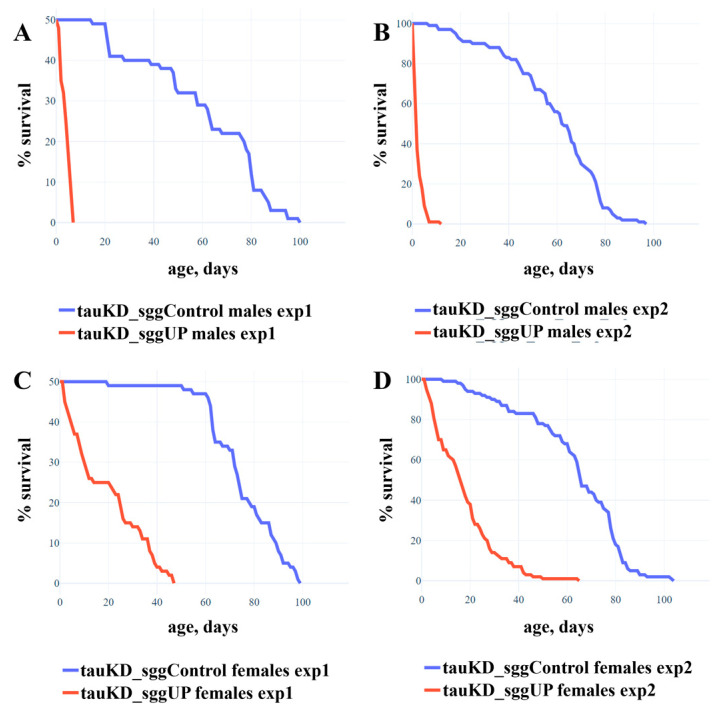
Co-activation of transgenes that decrease the expression of the *tau* gene and increase the expression of the *RB* transcript of the *shaggy* gene reduces the lifespan of males and females. (**A**,**B**)—Experiments 1 and 2 with males. (**C**,**D**)—Experiments 1 and 2 with females. A detailed description of the genotypes is given in the Materials and Methods section. Statistical analysis of the data is given in the Supplementary Materials Table S4.

In two repeated experiments, the lifespan of males with decreased *tau* expression and increased *shaggy* expression was very low compared to controls (Figure 4A,B; mean lifespan ~3 days) and did not exceed that of males with increased *shaggy* expression ([54], mean lifespan ~2 days). In two repeated experiments, the lifespan of females with decreased *tau* expression and increased *shaggy* expression was greatly reduced compared to controls (Figure 4C,D; mean lifespan ~18 days) but increased three-fold compared to that of females with increased *shaggy* expression ([54], mean lifespan ~6 days).

Experiments showed that in males, decreasing the amount of tau protein did not mitigate the effect of excess GSK3 on lifespan. This result does not suggest that the tau protein is a target of GSK3 protein kinase in the male lifespan control pathway. However, in females, the decrease in tau protein mitigated the effects of excess GSK3 on lifespan, which, in accordance with our hypothesis, suggests that the tau protein is a target of GSK3 protein kinase in the female lifespan control pathway. Thus, this result shows for the first time that in females, tau and GSK3 interact in lifespan control. The causes of the differences in the control of the lifespan of individuals of different sexes remain unclear. In particular, it is unclear whether in males, tau and GSK3 do not interact in the lifespan control in this case. It cannot be excluded that, in males, tau levels are reduced less than in females and that the amount of remaining protein is sufficient for the full implementation of GSK3 effects on longevity. It is possible that in males, the minor PE tau isoform that was not affected by *tau* knockdown is more involved in the interaction with GSK3. The involvement of the minor isoform can also explain the fact that, even in females, the *tau* knockdown did not fully restore the lifespan reduced by GSK3 excess. However, it is likely that the latter fact can be explained by the existence of other GSK3 targets and non-tau lifespan control pathways in which the protein kinase is involved, since its range of interactions is known to be very broad [57].

Another question arises as to why the interaction between tau and GSK3 could be observed only with a simultaneous decrease in *tau* expression and increase in *shaggy* expression but not with a simultaneous increase in the expression of both genes. It is possible that a decrease in *tau* expression actually reduced the amount of phosphorylated protein to mediate the negative effect of GSK3 overexpression on lifespan, whereas an increase in the *tau* expression in our experiments did not result in a critical increase in target molecules. Given the selective effect of knockdown and overexpression on the individual transcripts of the two genes, it is also possible that the observed phenomena could be explained by a complex interplay between the different isoforms of the two proteins.

### 2.2. Tau Affects the Properties of the Nervous System

The observed changes in lifespan were detected as a result of transgene activation in the nervous system, which allowed us to hypothesize that sex-dependent changes in lifespan may be accompanied by specific changes in the nervous system. It is known that the level of the phosphorylation of *tau* protein by GSK3 protein kinase is one of the key factors in the development of pathological changes in the structure and function of nervous tissue [58,59]. Previously, we showed that alterations in GSK3 activity in all neurons caused pathological changes in the morphology and function of the neuromuscular junctions of *Drosophila* larvae [54]. We hypothesized that these effects are mediated by the influence of GSK3 on *tau*, and therefore alterations in the expression of *tau* protein itself may lead to similar changes in the nervous system.

#### 2.2.1. Overexpression of the *RA* Transcript of the *Tau* Gene Mutant for GSK3 Phosphorylation Sites Affects Synaptic Activity in Females

To test the hypothesis that a sex-dependent change in lifespan induced by increased *tau* expression may correlate with changes in synaptic activity, we estimated the number of Brp spots in synaptic contacts in the neuromuscular junctions of males and females with the overexpression of the normal *RA* transcript of the *tau* gene and with the overexpression of the *RA* transcript of the *tau* gene mutant for GSK3 phosphorylation sites using the tauUP, tauUPmut, and tauUP-Control lines with the same genetic background. Transgene activation was induced in the nervous system at all stages of development using the pan-neuronal driver line NS. The number of active zones of synaptic contacts characterized by the number of spots of Bruchpilot (BRP), a protein specific to active synaptic zones, is often used to indirectly evaluate synaptic activity [60,61].

**Figure 5:**
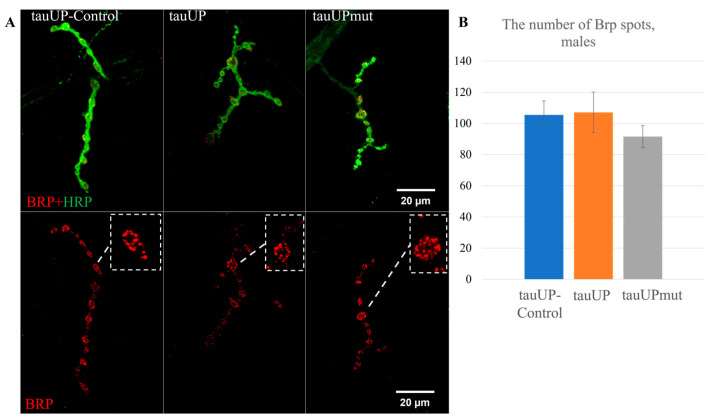

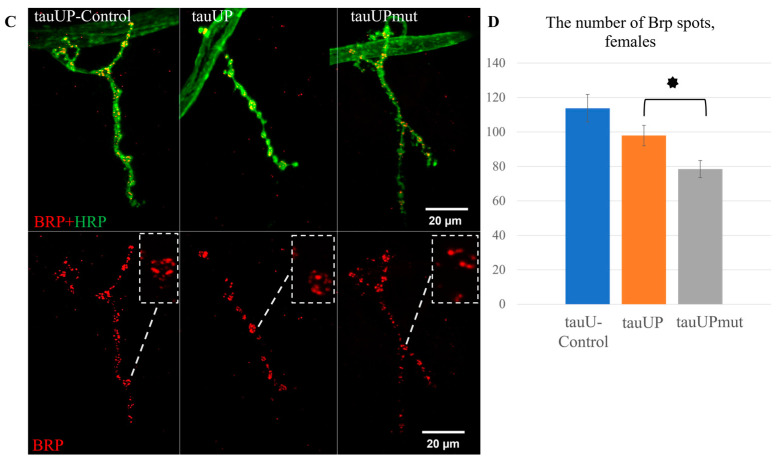
Increased expression of the normal and mutant *RA* transcript of the *tau* gene does not change the number of Brp spots in synaptic contacts in males and decreases it in females. (**A**,**B**)—Immunostaining and the mean number of Brp spots in males. (**C**,**D**)—Immunostaining and the mean number of Brp spots in females. A detailed description of the genotypes is given in the Materials and Methods section. * *p* < 0.05.

In males with an activated transgene carrying a normal or mutant copy of the *RA* transcript of the *tau* gene, the average number of BRP spots in synaptic contacts did not differ significantly from the control values (Figure 5A,B). This result is consistent with the negligible effect on lifespan observed in males of this genotype described above. Contrary to our initial hypothesis, in females with an overexpression of the normal *tau* transcript, there was no significant change in the average number of BRP spots in synaptic contacts compared to the controls (Figure 5C,D). Therefore, a significant change, namely a decrease in the number of Brp spots in synaptic contacts in females with an overexpression of mutant *tau* (*p* = 0.035, Figure 5D), was all the more surprising.

In our experiments, the increased expression of the normal *tau* gene in the nervous system did not affect the number of Brp spots in synaptic contacts. These data are consistent with the results showing that overexpression of the human *tau*, although it causes the impairment of endo- and exocytosis, does not change the active zones of synaptic contacts in neuromuscular junctions [62]. However, the expression of an additional copy of *tau* encoding a protein with disrupted GSK3 phosphorylation sites led to the impairment of the properties of the nervous system, namely a decrease in synaptic activity evaluated by the number of BRP spots, and only in females. We have already mentioned the fact, important in this context, that some tauopathies, such as Alzheimer’s disease, are significantly more common in women [52,53].

We selected the sites for mutations in the *tau* gene based on the homology of *Drosophila* tau regions with human tau regions associated with the pathologies of the nervous system. The nervous system impairment we detected, however, was not due to the increase in tau phosphorylation, as is supposedly the case in the development of human tauopathies [47,48,49,63,64,65,66]. In *Drosophila* with impaired synaptic activity, the amount of tau protein was increased, but its phosphorylation level did not exceed that of the control. On the contrary, an additional protein with impaired phosphorylation sites should have been phosphorylated to a lesser degree than normal. Consequently, we are not discussing the effect of an increased amount but the effect of conformational changes of this excessive isoform. Thus, the epitopes of the *Drosophila* tau, which correspond to the epitopes of the human tau associated with the pathologies of the nervous system, are functionally important in *Drosophila* and are also relevant to the nervous system. However, in *Drosophila* and humans with an impaired nervous system, the level of phosphorylation of these epitopes is probably different.

Interestingly, the changes in lifespan caused by the overexpression of the normal *Drosophila tau* were not associated with changes in the number of Brp spots in synaptic contacts. The lack of a link between survival and the nervous system is also confirmed by the fact that lifespan changed equally in flies with unchanged and altered synaptic activity.

#### 2.2.2. Overexpression of the *RA* Transcript of the *Tau* Gene Does Not Affect the Distribution of the Microtubule-Associated Futsch Protein

**Figure 6:**
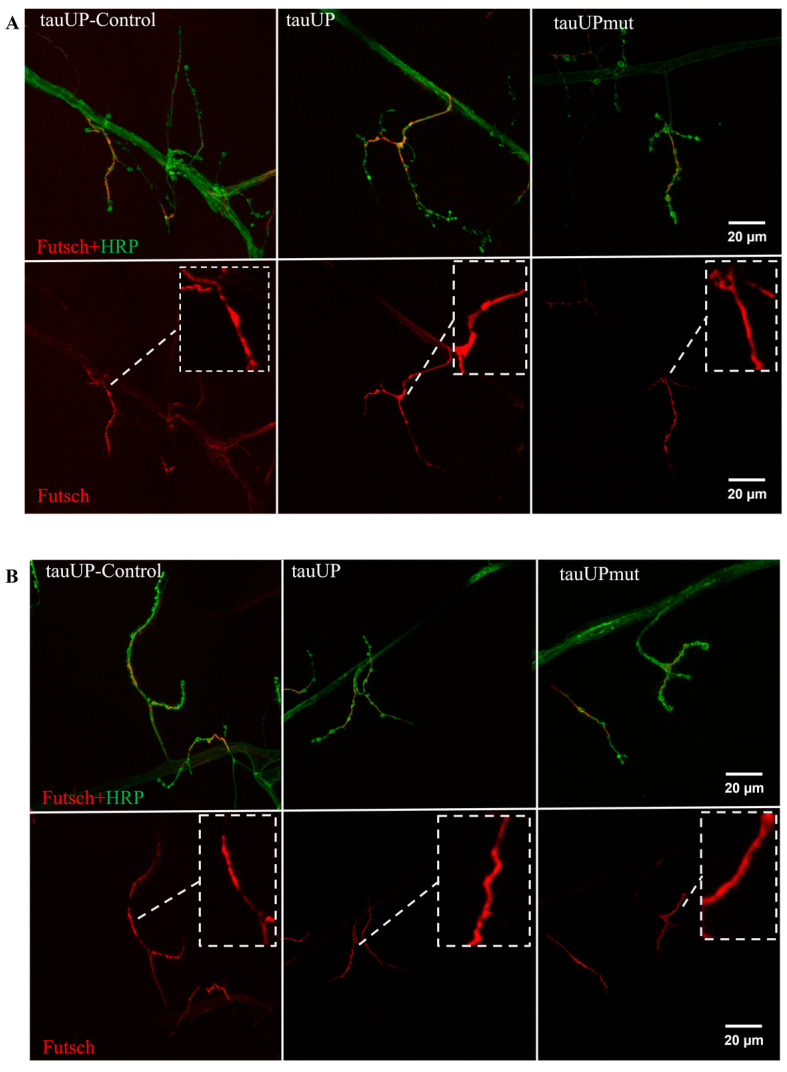
Increased expression of the normal and mutant *RA* transcript of the *tau* gene does not change the distribution of Futsch protein in males and females. (**A**)—Immunostaining in males. (**B**)—Immunostaining in females. A detailed description of the genotypes is given in the Materials and Methods section.

One of the possible causes of the impaired synaptic activity and disrupted transmission of components in the active zones of synaptic contacts may be a dysregulation of the axonal transport [17]. The tau protein belongs to a group of MAPs involved in the development of a wide range of neurodegenerative diseases [67,68]. In addition to tau, in *Drosophila*, other MAPs, such as Futsch, the human MAP1B homologue, are involved in the maintenance of cytoskeleton organization. Futsch colocalizes with microtubules and is required for cytoskeleton formation during axon growth and synaptogenesis. Futsch, like tau, is the target of phosphorylation by the protein kinase GSK3 [69]. Coordination in the shaping of certain cytoskeletal changes has been shown for the tau and Futsch proteins [70]; consequently, tau and Futsch can compensate for each other’s dysfunction. We analyzed the distribution of the Futsch protein in the neuromuscular junctions of males and females with an activated transgene encoding either the normal or the mutant *RA* transcript of the *tau* gene using the tauUP, tauUPmut, and tauUP-Control lines with the same genetic background. Transgene activation was induced in the nervous system at all stages of development using the pan-neuronal driver line NS. In none of the cases studied were males and females with *tau* overexpression found to differ significantly from individuals of the corresponding control genotypes in the distribution of the Futsch protein (Figure 6A,B).

The activation of expression of the transgene encoding the human *tau* gene in *Drosophila* is known to affect microtubules and, in particular, the Futsch protein [71]. However, in our model, the distribution of the MAP-related Futsch protein did not differ from the control in both sexes when either normal or mutant *RA* transcript of the *tau* gene was overexpressed. It is likely that the lack of effect may be due to the fact that different tau protein isoforms may interact differently with the Futsch protein.

The absence of irregularities in the Futsch distribution in individuals with an overexpression of mutant *tau*, demonstrated in this subsection of the results, suggests that the decrease in the number of Brp spots in synaptic contacts in animals of the same genotype described above was not related to changes in the cytoskeleton. It can be assumed that, in this case, the effect of the mutant *tau* overexpression on synaptic activity is not related to the canonical function of *tau*, which is to maintain microtubule stability.

#### 2.2.3. Knockdown of the *Tau* Gene Does Not Affect Synaptic Activity Reduced by the Overexpression of the *RB* Transcript of the *Shaggy* Gene

**Figure 7:**
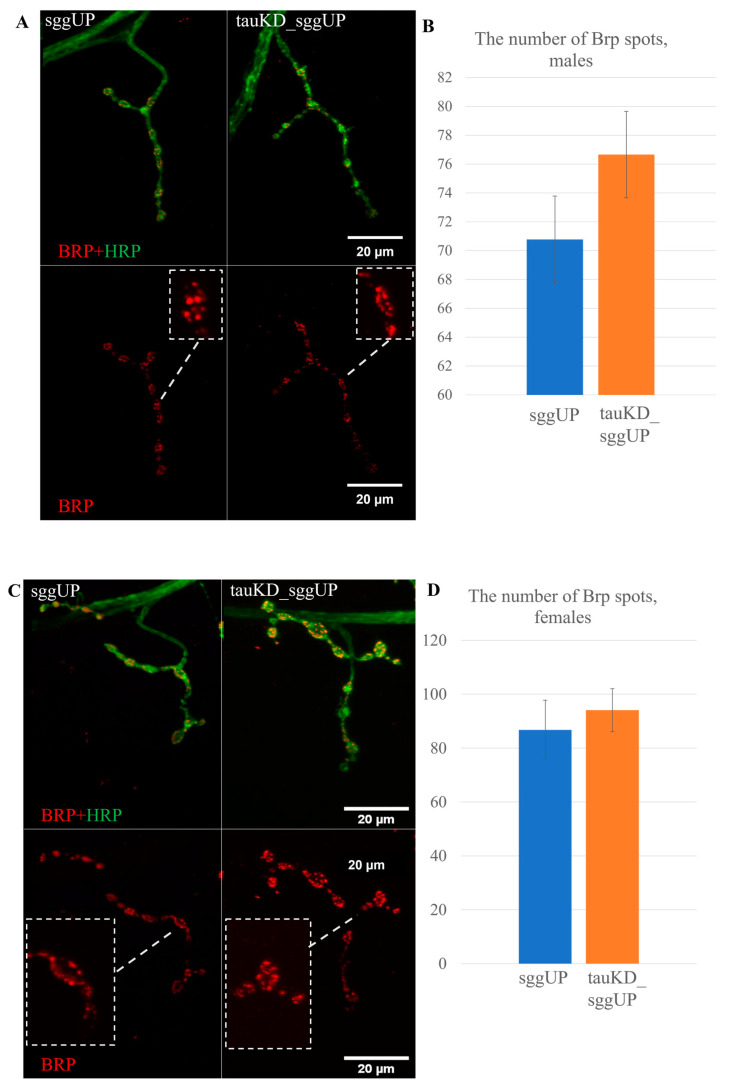
Activation of the transgene that decreases the expression of the *tau* gene does not affect the number of Brp spots in synaptic contacts reduced by the activation of the transgene that increases the expression of the *RB* transcript of the *shaggy* gene. (**A**,**B**)—Immunostaining and the mean number of Brp spots in males. (**C**,**D**)—Immunostaining and the mean number of Brp spots in females. A detailed description of the genotypes is given in the Materials and Methods section.

It was shown in [72] that the deletion of the *tau* gene in the fruit fly positively affected the number of zones of active synaptic contacts in neuromuscular junctions, reduced in models of neurodegenerative diseases caused by the expansion of trinucleotide repeats. We previously demonstrated the negative effect of the overexpression of the *RB* transcript of the *shaggy* gene in the *Drosophila* nervous system on the number of zones of active synaptic contacts [54]. We hypothesized that, in this case, the decrease in the amount of tau protein resulting from *tau* knockdown may also have a positive effect on the number of Brp spots in synaptic contacts. To test this assumption, we compared the number of Brp spots in synaptic contacts in individuals with a co-activation of the transgenes that decreased the expression of the *tau* gene and increased the expression of the *RB* transcript of the *shaggy* gene and in individuals with the activation of the transgene that increased the expression of the *RB* transcript of the *shaggy* gene using the tauKD_sggUP and sggUP lines. Transgene activation was induced in the nervous system at all stages of development using the pan-neuronal driver line NS. The overexpression of the *shaggy* gene decreased the number of Brp spots in synaptic contacts compared to controls, which is consistent with our earlier data. The knockdown of the *tau* gene in the background of *shaggy* overexpression did not result in a significant change in the number of Brp spots in synaptic contacts in either males or females (Figure 7A–D).

Knockdown of the *tau* gene in the nervous system, which does not affect the lifespan of males and females per se, positively affected the significantly reduced lifespan of females with a pan-neuronal increase in GSK3 expression. However, it did not affect the reduced number of Brp spots in the synaptic contacts of these females. This result further confirms that changes in lifespan and the properties of the nervous system do not always have common molecular causes. Our data may also indicate that tau is not the main target of GSK3 in the process of the formation of synaptic active zones, and changes in their number may occur due to alternative GSK3 targets. For example, it is known that the activation of GSK3 in mice reduces the expression of synapsin I, a protein of synaptic vesicles. Such a mechanism may underlie memory disorders associated with GSK3 activity [73]. At the same time, a comparison of our results with those demonstrated in [72] shows that the interaction of proteins is in fact based on the interaction of their different isoforms. The functions of the different protein isoforms may have been affected by the deletion [46] and knockdown of *tau*. It also cannot be ruled out that the decrease in *tau* expression caused by RNA interference may not have been sufficient to produce an effect comparable to that of the deletion of all exons with predicted microtubule binding sites.

#### 2.2.4. Knockdown of the *Tau* Gene Positively Affects the Distribution of the Microtubule-Associated Futsch Protein Impaired in Females with the Overexpression of the *RB* Transcript of the *Shaggy* Gene

**Figure 8:**
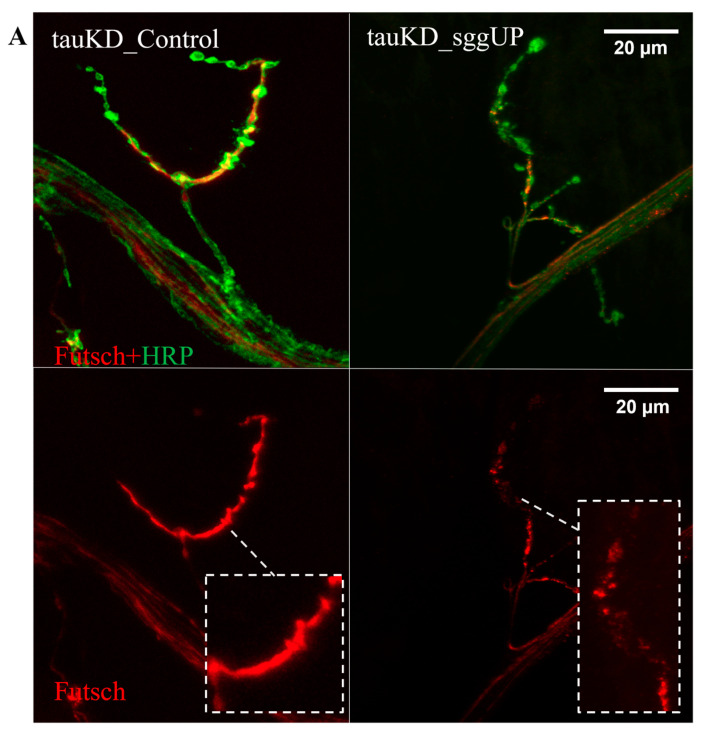

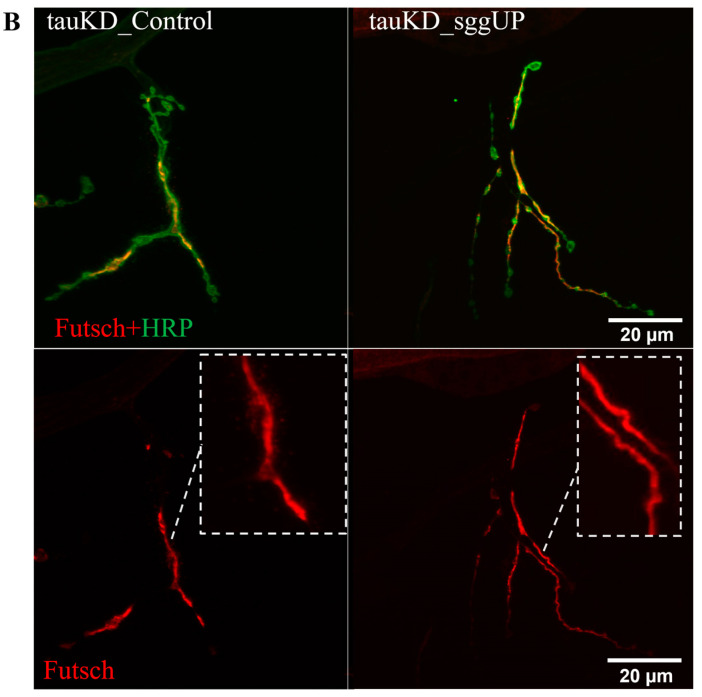
Activation of the transgene that decreases the expression of *tau* gene restores the distribution of Futsch protein impaired by the activation of the transgene that increases the expression of the *RB* transcript of the *shaggy* gene in females. (**A**)—Immunostaining in males. (**B**)—Immunostaining in females. Futsch is impaired by the *shaggy* overexpression in females but not in males. A detailed description of the genotypes is given in the Materials and Methods section.

Previously, we demonstrated that the increased expression of the *RB* transcript of the *shaggy* gene in the nervous system of male and female *Drosophila* altered the distribution of the Futsch protein in the synaptic boutons of neuromuscular junctions [54]. Since tau and Futsch can compensate for each other’s dysfunction [70], we analyzed the distribution of the Futsch protein in the neuromuscular junctions of males and females with activated transgenes causing the knockdown of *tau* gene and the overexpression of the *RB* transcript of the *shaggy* gene using the tauKD_sggUP and tauKD_Control lines. The transgene activation was induced in the nervous system at all stages of development using the pan-neuronal driver line NS. In males, the immunohistochemical staining of neuromuscular junctions revealed significant changes in the distribution of Futsch when compared with controls (Figure 8A). In females, no significant differences in the distribution of the Futsch protein from the control were detected (Figure 8B). Thus, a decrease in *tau* expression in females restored the disturbed distribution of Futsch caused by an increase in *shaggy* expression.

It remains unclear which molecular mechanism may be responsible for the effect of *tau* expression on the distribution of Futsch. It can be assumed that the phosphorylation level and stability of the Futsch protein, a direct target of the GSK3 protein kinase, may vary, depending on the total expression of the proteins of the MAP family. However, in this case, an increase rather than a decrease in the amount of *tau* against the background of GSK3 excess would lead to a compensatory effect, which we did not observe in our experiments. In [46], it was demonstrated that the *tau* deletion does not trigger compensatory mechanisms affecting the mRNA expression of other genes encoding MAPs, including Futsch; however, in that case, GSK3 expression remained normal. So far, data on the effect of reduced *tau* expression on the Futsch protein are very scarce, and this issue requires further study.

Of note, in females, *tau* knockdown against the background of increased *shaggy* expression leads to the compensation of the negative effect of GSK3 excess on both Futsch distribution and survival in females. Therefore, in this case, the change in *tau* and GSK3 expression has a coordinated effect on the properties of the nervous system and lifespan.

## 3. Materials and Methods

### 3.1. Lines and Crossings

The sggUP line, *w [1118]; P{w + mC = UAS-sgg. B}MB5,* containing the transgenic construct that encodes the normal PB form of GSK3 was used to provide sgg-RB overexpression upon induction; the co-isogenic sggUP-Control line, *w [1118]*, without any transgenic construct, was used as a control line. The lines described in [74] were obtained from Bloomington Drosophila Stock Center, USA. Flies of the sggUP line have red eyes because *w [1118]* does not show up due to insertion, and flies of the sggControl line have white eyes, which has little or no effect on lifespan [75].

To obtain lines providing *tau-RA* overexpression, cDNA corresponding to this transcript was cloned into the *pBID-UASC* vector (Addgene, Watertown, MA, USA, https://www.addgene.org), which contains an attB site for the phi31 site-specific transformation of Drosophila embryos and a UAS enhancer sequences [55]. One clone with intact tau-RA sequences was sent to Evrogen (Moscow, Russia, https://evrogen.com) for in vitro mutagenesis at sites selected according to the assay described below. The original clone with the normal *tau-RA* sequence, the clone with the mutant *tau-RA* sequence, and the line y1 M{vas-int.Dm}ZH-2A w*; M{3xP3-RFP.attP’}ZH-51C with the second chromosome attP phi31 integration site [56] were then used for the transformation performed by BestGene, Inc. (Chino Hills, CA, USA., https://www.thebestgene.com/HomePage.do). In the transgenic lines obtained, most of the auxiliary sequences were excised from the inserted transgenes due to the induction of recombination between the loxP sites present in the pBID-UASC vector.

The tauUP and tauUPmut homozygous transgenic lines, y [1] M{vas-int.Dm}ZH-2A w*; M{3×P3-RFP.attP’}ZH-51C P{w + mC = UAS-*tau*.A}2M and y1 M{vas-int.Dm}ZH-2A w*; M{3×P3-RFP.attP’}ZH-51C P{w + mC = UAS-*tau*.A[mut]}2M, containing the transgenic constructs that encode the normal and mutant PA form of Tau, respectively, were used to provide *tau-RA* overexpression upon induction. The tauUP-Control line, y1 M{vas-int.Dm}ZH-2A w*; M{3xP3-RFP.attP’}ZH-51C, initially used for transformations, was used as a control line with the same genetic background. Flies of all three lines have yellow bodies and white eyes.

The tauKD line, *y [1] v [1]; P{y[+t7.7] v[+t1.8] = TRiP.HMS02042}attP40*, containing the transgenic construct that encodes dsRNA complementary to *tau* mRNA was used to provide an RNAi knockdown of endogenous fruit fly *tau* upon induction and the co-isogenic tauKD-Control line, *y [1] v [1]*; *P{y[+t7.7] = CaryP}attP40*, without the transgene encoding dsRNA was used as a control line, as suggested by the manufacturer (DRSC/TRiP Functional Genomics Resources, Boston, MA, USA., https://fgr.hms.harvard.edu/). Previously, the authors of [50] demonstrated a significant decrease in the amount of protein. Flies of the tauKD and the tauKD_Control lines have red eyes of various shades. The *y [1]* mutation, which was shown to be long lived [75], does not appear in either line.

To analyze the interaction between the tau and shaggy genes, we used standard substitution crosses with balancers to obtain lines with the first chromosome marked with the *w [1118]* mutation from the sggUP-Control line; the second chromosome with transgenes, providing tau overexpression or knockdown from the tauUP, tauUPmut, or tauKD lines; and the third chromosome providing shaggy overexpression from the sggUP line or the control third chromosome from the sggUP-Control line. In total, the following lines were obtained: tauUP_sggUP, tauUPmut_sggUP, and tauUP_sggUP-Control; tauKD_sggUP, and tauKD_sggUP-Control.

To induce the expression of the transgenic constructs, females from the pan-neuronal driver line NS, *P{w[+mW.hs] = GawB}elav[C155] w [1118]; P{w[+mC] = UAS-Dcr-2. D}2*, obtained from the Bloomington *Drosophila* Stock Center were crossed with males from the sggUP, tauUP, tauUP-mut, tauKD, tauUP_sggUP, tauUPmut_sggUP, tauKD_sggUP, or the corresponding control lines. In all tested individuals, including control flies, the same GAL4 driver was present in all experiments, which allowed us to account for the effect of the insertion of the transgene encoding GAL4 on the traits under study. The GAL4 transgene came from females, which also accounted for the maternal effect. In control flies, the chromosomes with the UAS transgenes were absent; instead, the original chromosomes without transgenes, which were used for insertion, were present. The attP *phi31* integration site was initially embedded in these control chromosomes and further used for transgene insertions. Thus, the same disruption of the original chromosomal structure was present in both control and experimental lines. This design allowed us to account for the possible effect of the chromosomal disruption per se on lifespan and other phenotypes.

Flies were kept at 25 °C on a standard medium of semolina, sugar, raisins, yeast, and agar with nipagin, propionic acid, and streptomycin.

### 3.2. Tests for Wolbachia

All lines were tested for the presence of the *Drosophila* symbiont *Wolbachia* [76], using a quantitative PCR (MiniOpticon real-time PCR detection system, Bio-rad, (Hercules, CA, USA, https://www.bio-rad.com/)) with primers for the 16S rRNA gene, 5′-CATACCTATTCGAAGGGATAG-3′, and 5′-AGCTTCGAGTGAAACCAATTC-3′ [77].

### 3.3. Lifespan Assay

Lifespan was measured as previously described in [78]. Five virgin flies of the same genotype and sex, all collected on the same day from cultures with moderate density, were placed in replicate vials. Flies were transferred to vials with fresh food weekly. The number of dead flies was recorded daily. Experiments comparing fly lifespans were conducted simultaneously. Sample sizes were 50 to 100 flies per sex per genotype. The experiments with noteworthy results were repeated with an interval of approximately six months. The lifespan for each fly was estimated as the number of days alive from the day of eclosion to the day of death. Mean lifespans and survival curves were primarily used to characterize lifespan.

### 3.4. Immunostaining and Microscopy

Male and female third instar larvae ready for pupation were dissected in phosphate-buffered saline (PBS), fixed in 4% paraformaldehyde (Sigma-Aldrich, St. Louis, MO, USA, https://www.sigmaaldrich.com) at room temperature for 20 min, then washed in PBS (3 × 15 min). For immunostaining, preparations were blocked in 4% Normal Goat Serum for one hour at room temperature. Primary antibody incubation was performed overnight at 4 °C, washed in PBS (3 × 15 min), incubated in secondary antibodies for two hours, washed in PBS (5 × 10 min), and placed in a medium for immunofluorescence (VectaShield, Vector Labs, Newark, CA, USA, https://vectorlabs.com/). Neuromuscular junctions were analyzed in the fourth muscle of the third and fourth abdominal segments of larvae. A confocal laser scanning microscope (LSM 900, Zeiss, Jena, Germany, https://www.zeiss.com/), ImageJ 1.53 (http://rsb.info.nih.gov/ij/index.html) and ZEN Blue 3.6 (Zeiss) software were used. Sample sizes were 10 to 15 specimens per genotype per experiment.

The following primary antibodies were used: mouse anti-BRP (mAb NC82, 1:200; Developmental Studies Hybridoma Bank (DSHB, Iowa, IA, USA, https://dshb.biology.uiowa.edu/)) against Bruchpilot (BRP), a protein specific to active synaptic zones and required for vesicle release [60,61]; mouse anti-Futsch (mAb 22C10, 1:200; DSHB) against the microtubule-associated protein Futsch [79]; and Alexa Fluor 488-conjugated goat anti-HRP (1:500, Jackson ImmunoResearch, West Grove, PA, USA, https://www.jacksonimmuno.com/), against Horseradish Peroxidase (HRP), a widely used marker of presynaptic membranes [80]. Goat anti-mouse Cy3 conjugated (1:500, Jackson ImmunoResearch) were used as the secondary antibodies. Antibodies obtained from the DSHB were developed under the auspices of the NICHD and maintained by the University of Iowa, Department of Biology, Iowa City, IA 52242. The mean number of BRP spots in neuromuscular junctions was used to indirectly characterize synaptic activity. Futsch phenotype was qualitatively rated by the protein distribution uniformity inside of neuromuscular junctions.

### 3.5. Phosphorylation Sites and Mutagenesis

For the mutagenesis of GSK3-associated phosphorylation sites in the *D*. *melanogaster* tau protein, we chose the PA isoform because it corresponds to the major transcript of the *tau* gene [81] and includes all four microtubule binding sites that exist in the human tau. We compared the sequence of the PA isoform of the fruit fly tau (https://flybase.org/reports/FBtr0344741, accessed on 20 January 2023) with the longest 2N4R isoform of the human tau (https://www.ncbi.nlm.nih.gov/protein/NP_005901.2, accessed on 20 January 2023).

The analysis of the available data allowed us to identify a number of phosphorylation sites that were potentially associated with pathological changes in the human tau protein. Of these, we selected the following amino acid residues that are phosphorylated by the GSK3 beta protein kinase: S199, Thr231, and S 396 [47,49,66,82]. Next, using a multiple sequence alignment server (http://tcoffee.crg.cat, accessed on 20 January 2023), the amino acid sequence surrounding the selected human tau phosphorylation site was aligned with the corresponding amino acid sequence of D. melanogaster. The alignment resulted in the following matches:

S199 *H*. *sapiens*—S103 *D*. *melanogaster*.

Thr231 *H*. *sapiens*—Thr151 *D*. *melanogaster*.

S396 *H*. *sapiens*—S343 *D*. *melanogaster*.

The significance of the selected phosphorylation sites in the fruit fly tau protein was predicted using the bioinformatic resource NetPhos (https://services.healthtech.dtu.dk/service.php?NetPhos-3.1, accessed on 20 January 2023, predictive confidence threshold > 0.5). In the course of cloning the *Drosophila* tau gene (see details in Section 3.1), in order to mask the target phosphorylation sites, serine and threonine in the three selected sites were replaced with alanine, which should not greatly alter the protein structure and cannot be phosphorylated.

### 3.6. Statistical Analyses

To compare the control and mutant genotypes, Student’s t-test and the nonparametric, distribution-free Kruskal–Wallis test were used for the analyses of the number of Brp spots in neuromuscular junctions. These two tests gave consistent results, so only the results of the Kruskal–Wallis test are reported here. The standard descriptive statistical analysis of lifespan [83,84] was performed to determine the mean lifespan and its accompanying variances, standard deviations, and standard errors; the median, minimum, and maximum lifespans; and the lifespans of the lower and upper quartiles and the 10 and 90 percentiles (Tables S1–S4, Supplementary Materials). Survival curves were estimated using the Kaplan–Meier procedure. The nonparametric, distribution-free Mann–Whitney test and the Kolmogorov–Smirnov test were used to evaluate the statistical significance of the difference between the survival curves.

## 4. Conclusions

Lifespan is one of the most important quantitative characteristics of living organisms. Closely associated with it, aging is accompanied by a multitude of functional changes in various tissues and organ systems [85]. The nervous system plays a key role in communication between other body systems and regulates the response to many external and internal factors. In this context, the increase in nervous system dysfunction observed at a later age can lead not only to neurodegenerative diseases [86,87], but also to aging, thus reducing lifespan. At the molecular level, the relationship between the dysfunctions of the nervous system and decreased longevity is manifested in the fact that the same genes and proteins control both processes.

Among the considerable number of proteins associated with the occurrence of diseases of the nervous system, the tau protein plays an important role [6]. Note, however, that studies on this protein have yielded many contradictory results. There is evidence that the level of tau activity can both promote and be a marker of nervous system diseases and play a protective role [88,89,90]. The revealed contradictions could be due to the fact that, in a number of cases, tau properties were studied without taking into account specific protein isoforms, the genetic environment, and the sex of individuals. As for the effect of tau on lifespan, it has not been sufficiently characterized [46].

**Figure 9:**
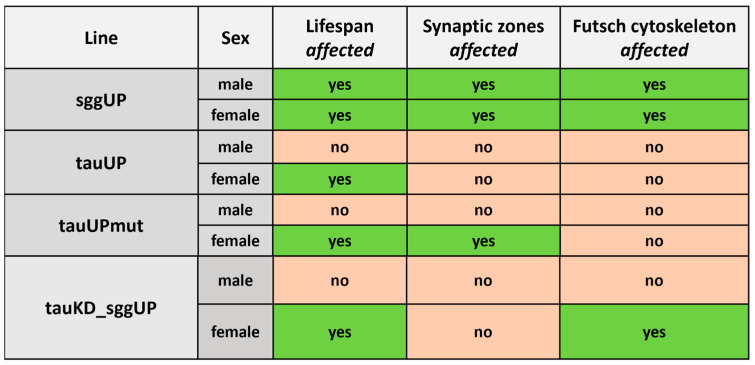
Effects of *shaggy RB* overexpression (according to [54]), *tau RA* overexpression, and *tau* knockdown in neurons on lifespan and the properties of the nervous system.

The data we obtained show that both an increase and a decrease in tau protein expression can affect lifespan and the properties of the nervous system and thus, in general, confirm the common genetic control of these traits (Figure 9). However, our results suggest that changes in lifespan and the specific properties of the nervous system as a result of impaired tau expression may be dissociated. In our model systems, changes in synaptic activity, which usually serve as a marker of the functional state of the nervous system, proved to be unrelated to changes in lifespan. In general, the observed pattern of effects depended on the direction of changes in tau expression, its interaction with other proteins, and the sex of the flies (Figure 9).

We confirmed the earlier data [46] that the loss of tau function does not lead to a change in lifespan. The lack of vital functions of tau in Drosophila may be partially related to the overlapping functions of other proteins belonging to the MAP family, such as Futsch. We showed that the effect of tau depletion depends on the expression level of one of the main tau regulators, the protein kinase GSK3. The reduction in tau expression mitigated the strong negative effect of GSK3 overexpression, not only on lifespan but also on Futsch properties. Thus, the mutual compensation of the functions of different MAPs may be important both for the control of nervous system properties and for the determination of longevity. Taken together, the data presented demonstrate the importance of the interaction of the tau protein with protein kinases, which phosphorylate it and thus regulate its activity, and with MAPs, which can compensate its functions.

We demonstrated that the effect of tau on lifespan and the properties of the nervous system depend on sex. On the one hand, this dependence can be explained by the fact that tau and/or GSK3 are exposed to proteins that directly regulate sex determination or to any sex-dependent general factors related, for example, to the insulin regulatory pathway [91]. On the other hand, this dependence can be explained by the specific functions of the individual isoforms of tau and GSK3 proteins. For example, the effect of the expression level of the RA transcript of the tau gene only on the lifespan of females may indicate the specific role of this particular isoform in individuals of that sex. Previously, we had also demonstrated that the effect of the increased expression of various shaggy transcripts on lifespan depends both on the tissue where it is altered and on sex [54]. Our results suggest the need for close attention regarding the specific roles of individual tau isoforms and the individual isoforms of its main regulators.

It is important to mention that the results obtained were based on changes in tau expression throughout the nervous system. The activity of the GSK3 protein kinase, one of the main regulators of tau, is known to be determined by phosphorylation. In the nervous system, the level of the phosphorylation of the key epitopes of GSK3 involved in the control of its activity depends on the types of neurons, as well as on the sex and age of individuals [92]. Previously, we demonstrated that changing the activity of the main isoform of GSK3 in motor and dopaminergic neurons led to a sex-dependent increase in lifespan, while changing its activity in other types of neurons had a negative effect [54,93]. These data suggest that the nature of the effects of GSK3 on its main targets in the nervous system, such as the tau protein, may also depend on neuronal type in a sex-dependent manner. In turn, different tau isoforms may also have specific functions in different types of neurons. Further study on the role of the expression levels of individual tau isoforms in different types of neurons will help to significantly expand our understanding of the patterns of neurodegenerative diseases and the control of lifespan.

## Data Availability

Not applicable.

## Supplementary Materials

The following supporting information can be downloaded at: https://www.mdpi.com/article/10.3390/ijms24032166/s1.
