# Supplementary material for: The Effect of the Tau Protein on D. melanogaster Lifespan Depends on GSK3 Expression and Sex
Source: Int J Mol Sci. 2023 Jan 21;24(3):2166. doi: 10.3390/ijms24032166 (PMC9916465; doi:10.3390/ijms24032166)
Supplement: Supplementary file 1 [file ijms-24-02166-s001.zip › ijms-2111946-supplementary.pdf]

Tables with descriptive statistics of lifespan measurements

Abbreviations: M-W: Mann-Whitney; K-S: Kolmogorv-Smirnov

experiment	N	mean	median	min	max	lower quartile	upper quartile	10%	90%	dispersion	std.deviation	std.error	M-W	K-S
**tauUp-Control males exp1**	50	55,92	58	7	100	49	68,75	13,8	80,2	476,9730612	21,83971294	3,088601824	0.388561	0.271914
**tauUP males exp1**	50	54,82	56	6	80	54	62	40,1	69,6	241,742449	15,54806898	2,198829002		
**tauUP males exp1**	50	54,82	56	6	80	54	62	40,1	69,6	241,742449	15,54806898	2,198829002	0.242137	0.039195
**tauUPmut males exp1**	50	56,52	62,5	10	85	51,25	74	15,8	75	412,9893878	20,32214033	2,873984648		
**tayUP-Control males exp2**	100	57,02	60	14	96	49	72,5	18,7	78,3	446,5046465	21,13065656	2,113065656	0.015896	0.211170
**tauUP males exp2**	100	50,2	54,5	6	94	39,25	70	13,8	75	492,6060606	22,19473047	2,219473047		
**tauUP males exp2**	100	50,2	54,5	6	94	39,25	70	13,8	75	492,6060606	22,19473047	2,219473047	0.512425	0.368188
**tauUPmut males exp2**	100	49,38	52	8	85	36	60	21,9	77,1	352,8844444	18,78521878	1,878521878		
**tauUP-Control females exp1**	50	74,16	80,5	8	97	65,5	85,75	46,4	92,2	369,6473469	19,2262151	2,718997414	0.005675	0.021708
**tauUP females exp1**	50	64,94	70,5	10	91	59	79	22,7	84,2	440,7922449	20,99505287	2,969148851		
**tauUP females exp1**	50	64,94	70,5	10	91	59	79	22,7	84,2	440,7922449	20,99505287	2,969148851	0.320551	0.178587
**tauUPmut females exp1**	50	61,88	69	14	87	60,25	74,75	23,5	82	435,4955102	20,86852918	2,9512557		
**tauUP-Control females exp2**	100	73,48	77,5	18	105	67	86	52,9	90,1	313,7874747	17,71404738	1,771404738	0.000000	0.000000
**tauUP females exp2**	100	52,46	62	8	94	31,75	71,25	14	77	575,6246465	23,99217886	2,399217886		
**tauUP females exp2**	100	52,46	62	8	94	31,75	71,25	14	77	575,6246465	23,99217886	2,399217886	0.362538	0.154839
**tauUPmut females exp2**	100	56,62	62	9	87	49	71,25	19	76	406,8238384	20,16987453	2,016987453		

Table S1. Statistical analysis for the lifespan measurement in species with pan neuronal overexpression of *tau* transgenes.

experiment	N	mean	median	min	max	lower quartile	upper quartile	10%	90%	dispersion	std.deviation	std.error	M-W	K-S
**tauUP_sggControl males exp1**	100	47,82	52	6	78	40	59	23,3	62	265,8056566	16,30354736	1,630354736	0.000000	0.000000
**tauUP_sggUP males exp1**	100	1,76	1	1	7	1	2	1	3	1,295353535	1,138135992	0,113813599		
**tauUP_sggUP males exp1**	100	1,76	1	1	7	1	2	1	3	1,295353535	1,138135992	0,113813599	0.110500	0.583009
**tauUPmut_sggUP males exp1**	100	2,07	2	0	7	1	2	1	4	2,005151515	1,416033727	0,141603373		
**tauUP_sggControl males exp2**	100	41,3	43,5	5	88	24	55	14,8	67	396,0505051	19,90101769	1,990101769	0.000000	0.000000
**tauUP_sggUP males exp2**	100	3,27	3	1	7	2	5	1	6	2,845555556	1,686877457	0,168687746		
**tauUP_sggUP males exp2**	100	3,27	3	1	7	2	5	1	6	2,845555556	1,686877457	0,168687746	0.195715	0.815415
**tauUPmut_sggUP males exp2**	100	2,96	3	1	8	2	4	1	5	2,564040404	1,601262128	0,160126213		
**tauUP_sggControl females exp1**	100	55,23	61	8	84	46	68,5	22	76	376,0980808	19,39324833	1,939324833	0.000000	0.000000
**tauUP_sggUP females exp1**	100	3,96	2	1	33	1	4	1	6,1	39,69535354	6,300424869	0,630042487		
**tauUP_sggUP females exp1**	100	3,96	2	1	33	1	4	1	6,1	39,69535354	6,300424869	0,630042487	0.631596	0.908411
**tauUPmut_sggUP females exp1**	100	2,57	2	1	8	1	4	1	5	3,318282828	1,821615445	0,182161544		
**tauUP_sggControl females exp2**	100	61,49	68	13	89	60	76	16,9	78,1	459,0605051	21,42569731	2,142569731	0.000000	0.000000
**tauUP_sggUP females exp2**	100	2,92	2	1	12	2	4	1	5	3,508686869	1,873148918	0,187314892		
**tauUP_sggUP females exp2**	100	2,92	2	1	12	2	4	1	5	3,508686869	1,873148918	0,187314892	0.668639	0.999999
**tauUPmut_sggUP females exp2**	100	2,78	2	1	8	2	4	1	5	2,658181818	1,630393148	0,163039315		

Table S2. Statistical analysis for the lifespan measurement in species with simultaneous pan neuronal overexpression of *tau* and *shaggy* transgenes.

experiment	N	mean	median	min	max	lower quartile	upper quartile	10%	90%	dispersion	std.deviation	std.error	M-W	K-S
**tauKD_Control males**	100	62,49	60,5	26	96	56	74,25	46,7	80	196,8584848	14,03062667	1,403063	0.986348	0.053902
**tauKD males**	100	58,77	67,5	8	94	45,75	77	12,9	87	608,9667677	24,67725203	2,467725		
**tauKD_Control females**	100	75,12	84	13	98	70	90,25	37	93	454,5713131	21,32067806	2,132068	0.011968	0.000120
**tauKD females**	100	74,35	77	13	105	69,75	83	59	88,1	229,9469697	15,16400243	1,5164		

Table S3. Statistical analysis for the lifespan measurement in species with pan neuronal knockdown of *tau* gene.

experiment	N	mean	median	min	max	lower quartile	upper quartile	10%	90%	dispersion	std.deviation	std.error	M-W	K-S
**tauKD_sggControl males exp1**	50	60,96	63,5	15	100	48,25	80	21,9	87,1	600,5289796	24,5056928	3,465628	0.000000	0.000000
**tauKD_sggUP males exp1**	50	4,26	4,5	1	7	2	6	2	7	3,624897959	1,903916479	0,269254		
**tauKD_sggControl males exp2**	100	58,91	62,5	7	97	48,25	75	30,4	79	392,4261616	19,80974916	1,980975	0.000000	0.000000
**tauKD_sggUP males exp2**	100	2,68	2	1	12	1	3	1	5	3,694545455	1,922120042	0,192212		
**tauKD_sggControl females exp1**	50	75,26	74	20	99	64	87	62	92,3	218,4412245	14,77975725	2,090173	0.000000	0.000000
**tauKD_sggUP females exp1**	50	19,86	17,5	2	47	6,5	33,75	2,9	39,1	215,0616327	14,6649798	2,073941		
**tauKD_sggControl females exp2**	100	63,47	66	9	104	53,75	79	30,8	83	430,8778788	20,7575981	2,07576	0.000000	0.000000
**tauKD_sggUP females exp2**	100	17,7	16,5	2	65	7	25	4	36	158,2727273	12,58064892	1,258065		

Table S4. Statistical analysis for the lifespan measurement in species with simultaneous pan neuronal knockdown of *tau* gene and *shaggy* overexpression.
